# Spontaneous Recovery of Active Shoulder External Rotation in Patients with Brachial Plexus Birth Injury

**DOI:** 10.1097/PRS.0000000000011877

**Published:** 2024-11-20

**Authors:** Maria Hyttinen, Henrikki Rönkkö, Pasi Paavilainen, Mika Helminen, Jarkko Jokihaara

**Affiliations:** Tampere, Finland; From the 1 Faculty of Medicine and Health Technology; 6 Faculty of Social Sciences, Health Sciences, Tampere University; 2 Division of Musculoskeletal Diseases; 4 Tays Research Services, Tampere University Hospital; 3 Pihlajalinna Hospital; 5 Wellbeing Services County of Pirkanmaa.

## Abstract

**Background:**

Brachial plexus birth injury (BPBI) occurs as a result of a difficult delivery. External rotation (ER) of the shoulder (ER) is usually one of the last movements that may recover. There is no consensus about the predicting factors for spontaneous recovery or the optimal timing for surgical treatment of ER in BPBI patients. The aim of this retrospective study was to describe spontaneous recovery of active ER and evaluate predicting factors for the recovery.

**Methods:**

The authors screened 562 patients and identified a consecutive cohort of 103 BPBI patients, who had no active ER at the age of 3 months. The authors systematically collected clinical data on recovery. In addition, the authors assessed whether early recovery of elbow flexion, shoulder abduction, or Narakas grade at 1 month predicts ER recovery.

**Results:**

Fifty-two patients (51%) spontaneously recovered ER, 44% of whom recovered by the age of 1 year, 83% by 1.5 years, 92% by 2 years, and 98% by 3 years. A breakpoint in the slope of the curve showing proportion of recovered patients occurred at 2 years of age. Recovery of active ER was significantly associated with early elbow flexion and Narakas grade at 1 month, but not with early active shoulder abduction.

**Conclusions:**

Most spontaneous recovery of ER in patients with BPBI occurs until 2 years of age, which thus can be considered a meaningful follow-up period for spontaneous recovery of ER. This information should be considered when making decisions about peripheral nerve transfer surgery to improve ER in BPBI.

**CLINICAL QUESTION/LEVEL OF EVIDENCE:**

Risk, III.

Brachial plexus birth injury (BPBI) is caused by traction during a difficult delivery. The incidence of BPBI varies from 0.9 to 5.1 per 1000 births,^1–3^ and BPBI usually affects the C5 and C6 roots.^4^ Of all BPBI patients, 66% to 92% recover completely.^1,5,6^ Based on the regeneration speed of axons, the time for complete neurologic recovery can be up to 3 years, while ongoing functional recovery is rarely reported after 3 years of age.^5^

It is commonly suggested that children who recover antigravity elbow flexion (EF) function later than 3 months of age are more likely to have worse shoulder function than those who recover EF earlier.^6–8^ Accordingly, absent EF function at 3 months of age is generally regarded to reflect insufficient spontaneous nerve recovery and to be an indication for early surgical brachial plexus reconstruction.^5,9,10^ However, EF often recovers early, whereas active external rotation (ER) of the shoulder is among the last movements to recover in BPBI.^1^ The probability of spontaneous recovery of ER over time is not known; thus, there is no consensus on the optimal timing for making a treatment decision about a possible distal nerve transfer operation (spinal accessory nerve to suprascapular nerve [SAN-SSN]) to activate ER.

The rationale for the study was to evaluate spontaneous recovery of active ER after BPBI. In addition, we assessed whether early EF, Narakas grade at 1 month, and shoulder abduction (ABD) are predictors for spontaneous recovery of active ER. These results can be used when making treatment decisions about surgical treatment for absent active ER.

## PATIENTS AND METHODS

**Fig. 1.**
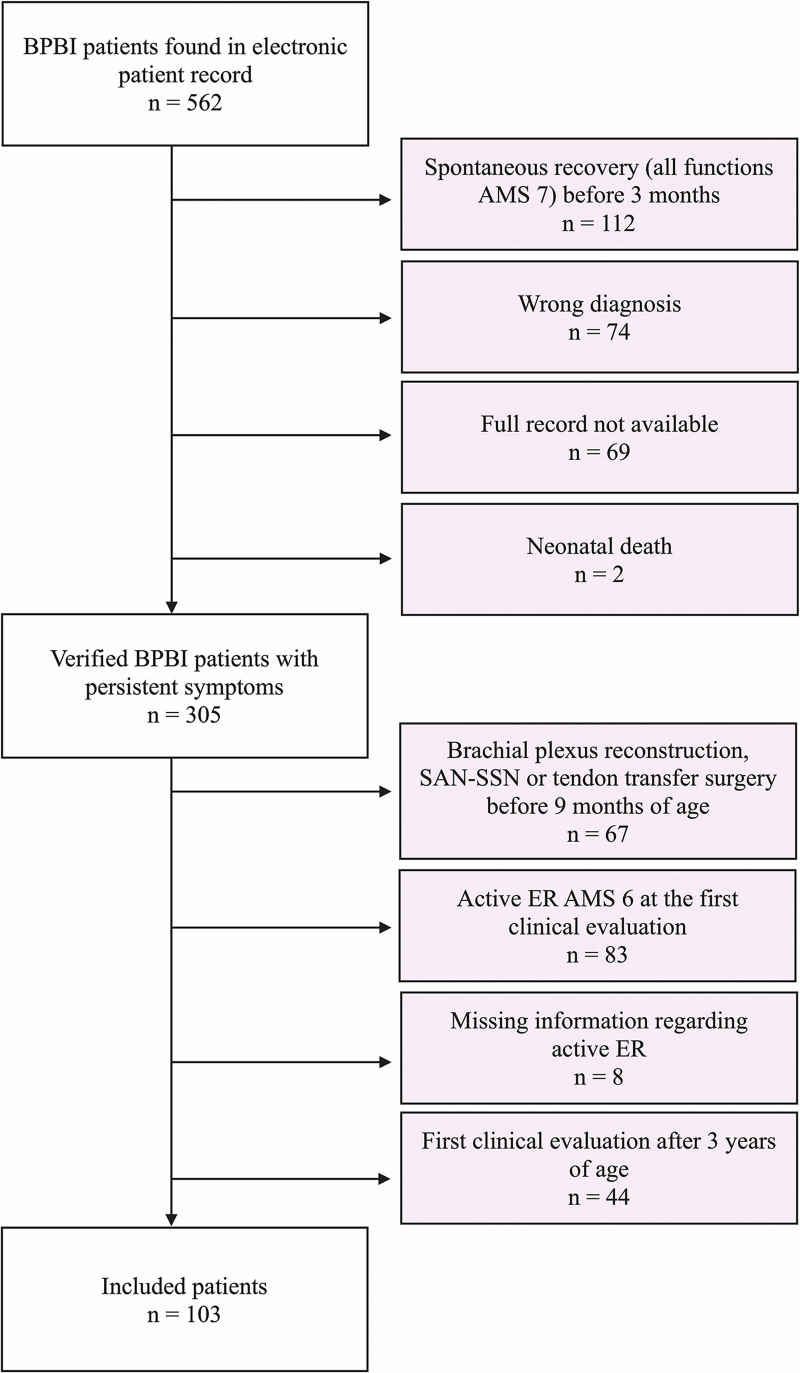
Flow diagram for inclusion and exclusion of patients.

In this retrospective study, we evaluated spontaneous recovery of active ER in BPBI patients who had no ER at the age of 3 months. The study conforms to the research guidelines of the study hospital ethical review board. The study results are reported according to the Strengthening the Reporting of Observational Studies in Epidemiology statement.^11^ To identify all potentially eligible patients, we screened children born between the years 2000 and 2021 from the electronic medical record with the diagnosis codes for birth injuries to the peripheral nervous system (*International Classification of Diseases, 10th Revision*, codes P14.0, P14.1, P14.2, P14.3, P14.8, and P14.9). From those (*n* = 562), we identified the patients who met the inclusion criteria of the study: (1) the patient had confirmed BPBI diagnosis, (2) the patient had not fully recovered (full recovery was defined as recovery of all upper extremity functions to grade 7 on the Active Movement Scale [AMS])^12^ within the first 3 months of life, (3) ER had been absent (AMS 0 to 5) at the clinical evaluation after 3 months of age, (4) the patient’s first clinical evaluation at the study hospital had been before 3 years of age, and (5) duration of follow-up for possible recovery was a minimum of 3 years. Patients who had undergone brachial plexus reconstruction or SAN-SSN transfer before 9 months of age were excluded, because these operations prevent spontaneous recovery of ER (Fig. 1). The data on patient and injury details and clinical findings during follow-up were collected from the patient record using the last observation carried forward because of some missing follow-up observations.^13^

The AMS is an 8-grade scale for evaluating motor function, and it can be used with children of any age. The scale ranges from 0 to 7, where AMS 0 means no muscle contraction, AMS 5 indicates active movement less than half the range of movement against gravity, AMS 6 means active movement at least half the range of movement against gravity, and AMS 7 means normal active movement.^12^ In our study, active ER, EF, and shoulder ABD at different time points were translated into AMS grade during the data collection. When the patient achieved ER AMS 6, in which the forearm reaches the sagittal plane (neutral position) with the arm in full adduction and the elbow in 90 degrees of flexion, the ER function was considered recovered.

Extent of BPBI was described with the Narakas classification at the age of 1 month.^14^ Evaluation of ER at follow-up assessments was performed and recorded independently by a hand surgeon specialized in BPBI and by a pediatric physiotherapist. Passive and active ER were evaluated in full shoulder adduction and elbow in 90 degrees of flexion. The presence of active ER was evaluated at the ages of 3 to 4 months, 5 to 6 months, 8 to 10 months, 1 year, 1.5 years, 1.75 years, 2 years, 2.25 years, 3 years, 5 years, and 7 years. The last evaluated follow-up visit took place at a mean age of 12.6 ± 2.4 years. The age when active ER improvement to AMS 6 was first observed was determined for every patient if such a recovery occurred.

The physiotherapy protocol used in our hospital consists of passive exercises started a few days after birth, sensory reeducation, and progressive strengthening of active movements. It consists of a weekly appointment with a physiotherapist and free passive range-of-motion exercises, particularly in shoulder ABD and ER, at home, according to the instructions given by the physiotherapist.

In our treatment protocol, patients with absent antigravity EF at 3 months are referred to surgical exploration of the brachial plexus. If there are viable nerve roots, reconstruction of brachial plexus with interposition nerve grafts is indicated. In case of upper root avulsions, nerve transfers are used (eg, SAN-SSN transfer) for ER. If early surgical treatment is not indicated and there is persistent deficiency in ER, we consider 1.5 years as a threshold for making a decision about SAN-SSN transfer. Tendon transfers (eg, latissimus dorsi and teres major tendon transfer to the infraspinatus tendon) have been used as later stage interventions in patients with absence of shoulder function; nowadays, they are seldom used because of the SAN-SSN transfer technique, which is the current standard practice.

All the patients included in the follow-up of spontaneous recovery had at least 70 degrees of passive ER. Botulinum toxin type A (BTX) injections to shoulder internal rotator muscles have been used at the study hospital since 2006. BTX injections are effective in maintaining the sufficient passive movement and shoulder congruency,^15^ and were administered to study patients if restriction of passive ER was observed before 1.5 years of age while waiting for the spontaneous recovery of active ER. Thus, patients treated in the beginning of the study period before the use of BTX injections were more likely to develop internal rotation contractures. Patients with passive restriction of ER or subluxation of the humeral head observed after 1.5 years of age are treated with anterior release surgery and subscapular tendon lengthening, if needed. Patients who underwent an anterior release surgery during the study period, were separated from the follow-up of spontaneous recovery.

Operative treatment for active ER (SAN-SSN transfer or a tendon transfer surgery) prevented further spontaneous recovery of the nerve and/or made evaluation of spontaneous recovery of ER impossible. Patients who underwent one of these procedures were identified and separated from the follow-up group at the time of the operation. The first SAN-SSN transfer surgery in our patient cohort took place in 2011; before that, if surgical treatment for active ER was indicated, patients were treated with tendon transfer.

A segmented linear regression analysis was conducted to find a transition point where a remarkable change occurs in the slope of the curve, representing cumulative percentage of recovered patients. As a planned sensitivity analysis, we evaluated the impact of estimation method for time point for recovery of ER to AMS 6. For that analysis, we identified and used the last clinical evaluation when ER AMS 6 was not yet observed in every recovered patient, because the true time point for recovery of ER to AMS 6 was between the last observation, when AMS was still less than 6, and the next, when AMS 6 was observed.

To evaluate the prognostic value of early EF and shoulder ABD when estimating recovery of ER, we divided the patients into groups based on their early EF (group EF 1, AMS 6 to 7 before 4 months of age; and group EF 2, AMS 0 to 5 at 4 months of age) and shoulder ABD (groups ABD 1 and ABD 2, respectively). The age limit was set at 4 months because we wanted to include the finding from the clinical appointment at the age of 3 months, which in practice may take place 1 to 2 weeks after the precise age of 3 months. For the analysis regarding association between the Narakas classification at 1 month and recovery of ER, we pooled Narakas 3 and 4 groups, as there was only 1 patient in group 4. To assess the impact of EF, shoulder ABD, and Narakas classification on recovery of ER, we used the Cox proportional hazards model. The patients who had undergone any surgery that may affect ER during the follow-up period, or did not recover active ER, were marked as censored observations.

For statistical analyses we used R, version 4.3.2 (R Foundation for Statistical Computing, Vienna, Austria) (package segmented^16^). Ages and recovery times are presented as mean with SD. The estimated breakpoint in the curve of recovered patients is reported with 95% CI and coefficient before and after the breakpoint, with SE, 95% CI for the coefficient, and the associated *P* value. The proportional hazards assumption was tested with the Schoenfeld residuals test, and crude and adjusted hazard ratios for the Cox proportional hazards regression are presented with 95% CIs and *P* values. (**See Table, Supplemental Digital Content 1**, which shows the Cox proportional hazards regression for spontaneous recovery of ER, http://links.lww.com/PRS/H688.) Analysis of variance *P* values are presented for each variable in the model. Kaplan-Meier hazard curves are shown with *P* values from log-rank tests. *P* < 0.050 was considered statistically significant.

## RESULTS

We identified 103 patients born between 2000 and 2021 with BPBI and missing active ER at 3 months of age. Patient characteristics are shown in Table 1.

**Table 1.** Patient Characteristics

Characteristic	No.
Sex	
Female	54
Male	49
Affected side	
Right	58
Left	45
Narakas classification at 1 mo	
Narakas 1 (C5, C6)	28
Narakas 2 (C5, C6, C7)	51
Narakas 3 (C5, C6, C7, C8, TH1)	8
Narakas 4 (C5, C6, C7, C8, TH1, Horner)	1
Not available	15
Elbow flexion at 4 mo	
AMS 0–5	50
AMS 6–7	45
Not available	8
Shoulder abduction at 4 mo	
AMS 0–5	55
AMS 6–7	15
Not available	33
Surgical treatment for ER	
SAN-SSN transfer	23
Tendon transfer for ER	12
Anterior release (all)	26
Humeral rotational osteotomy	4

### Recovery of ER

**Fig. 2.**
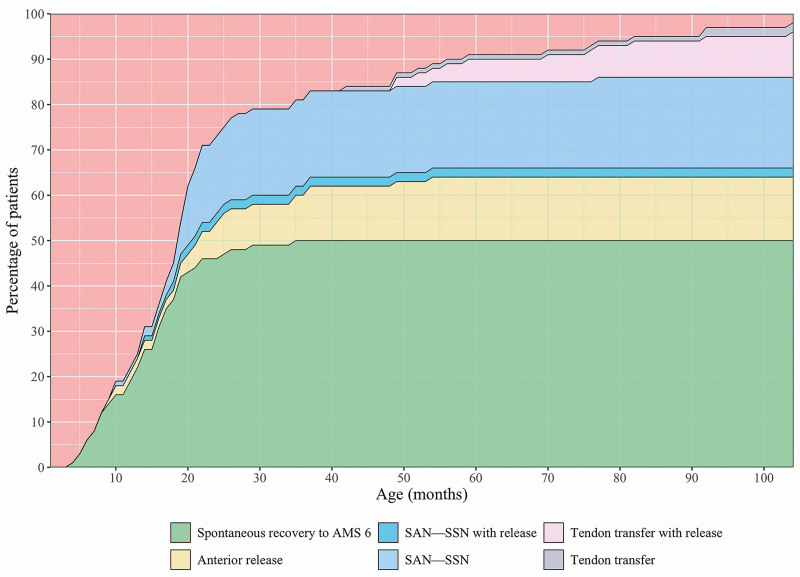
Recovery of active ER in 103 patients. The chart shows the cumulative percentage of patients who recovered ER spontaneously to AMS 6. Patients were excluded from the follow-up group if they underwent any of the following procedures: an anterior release operation with subscapular tendon lengthening if needed because of restricted passive ER, SAN-SSN transfer with or without simultaneous anterior release and subscapular tendon lengthening if needed, and a tendon transfer operation with or without simultaneous anterior release and subscapular tendon lengthening if needed.

Fifty-two of 103 patients (51%) spontaneously recovered active ER (Fig. 2). Of the 52 that recovered ER, 23 (44%) recovered by 1 year of age, 43 (83%) by 1.5 years, 48 (92%) by 2 years, and 51 (98%) by 3 years. The most significant breakpoint in the curve depicting the cumulative proportion of patients with spontaneous ER recovery was at the age of 22 months. Segmented regression analysis is presented in Table 2. In the confirmatory sensitivity analysis with differently defined time points for recovery, 45 of 52 patients (87%) would have been recovered by 1 year of age, 48 (92%) by 1.5 years, and 52 (100%) by 2 years.

**Table 2.** Segmented Linear Regression for Spontaneous Recovery of Active ER

Age Group	Breakpoint (mo)	95% CI (Breakpoint)	Coefficient	SE	95% CI (Coefficient)	*P* ^a^
Before age 22.36 mo	—	—	4.81	0.13	4.54–5.09	<0.001
After age 22.36 mo	22.36	21.61–23.12	0.31	0.04	0.23–0.38	<0.001

a Statistically significant.

Twenty-one patients underwent SAN-SSN transfer (at a mean age of 22 ± 12 months), and 2 underwent SAN-SSN transfer and anterior release with subscapular tendon lengthening by the same procedure at 14 and 18 months. Two patients underwent a tendon transfer at 42 and 92 months, and 10 patients underwent a tendon transfer with anterior release and possible subscapular tendon lengthening (mean, 69 ± 19 months). Fourteen patients, of whom 12 developed dorsal subluxation of the humeral head and glenohumeral dysplasia, ended up in an anterior release surgery after persistent restriction of passive ER (mean age, 33 ± 21 months) without any simultaneous surgery for active ER (Table 1). Eight of them recovered active ER after the release procedure, suggesting that without passive restriction the total proportion of patients with spontaneous recovery of active ER would have been larger.

Two patients did not recover active ER to AMS 6 and did not undergo any surgical treatment. Both of these patients had restriction of passive ER already in the first months of life. In six patients who spontaneously recovered ER to AMS 6, we observed deterioration of ER during the follow-up, at a mean age of 4.8 ± 3.8 years. Two of these patients developed dorsal subluxation of the humeral head and glenohumeral dysplasia, with 1 experiencing permanent deterioration leading to ER AMS 0. Five patients regained active ER AMS 6 after a period of weaker function.

### Relationship between Early EF and Recovery of ER

**Fig. 3.**
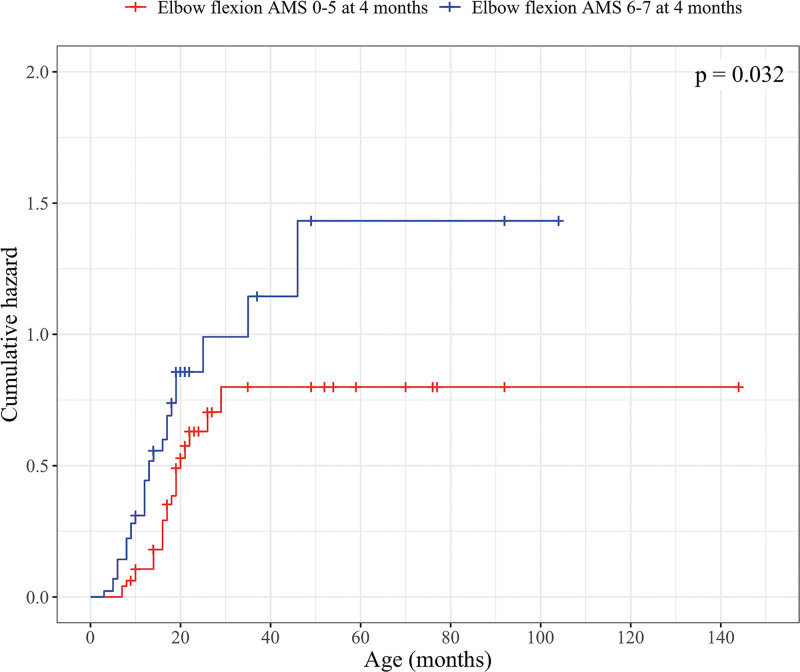
Kaplan-Meier hazard curves and log-rank test *P* value for active EF as a predictor for ER. The *blue line* indicates the accumulated probability of ER recovery in patients with AMS 6 to 7 EF before 4 months of age; the *red line* indicates the accumulated probability of ER recovery in patients with AMS 0 to 5 EF at 4 months of age. Patients who are censored either did not achieve recovery of ER to AMS 6 by the end of the follow-up or underwent any of the following procedures: an anterior release surgery with subscapular tendon lengthening if needed because of restricted passive ER, SAN-SSN transfer with or without simultaneous anterior release and subscapular tendon lengthening if needed, and a tendon transfer operation with or without simultaneous anterior release and subscapular tendon lengthening if needed.

Patients were divided into 2 groups based on their EF at 4 months: AMS 6 to 7 (group EF 1) and AMS 0 to 5 (group EF 2). Eight patients were excluded from this part of the analysis because of missing information regarding EF (Table 1). Sixty-two percent (28 of 45) of patients in group EF 1 and 46% (23 of 50) in group EF 2 spontaneously recovered active ER AMS 6. The groups EF 1 and EF 2 were significantly different in Kaplan-Meier analysis and univariate Cox regression (Fig. 3) (**see Table, Supplemental Digital Content 1**, http://links.lww.com/PRS/H688).

### Relationship between Early Shoulder ABD and Recovery of ER

**Fig. 4.**
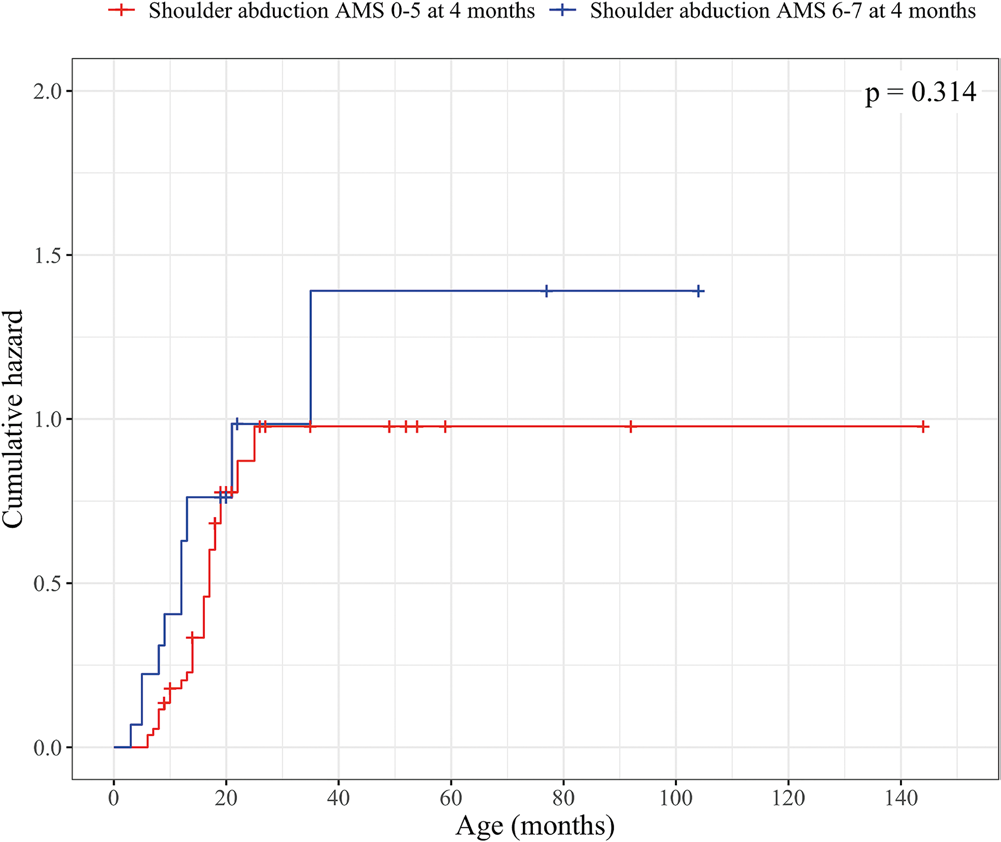
Kaplan-Meier hazard curves and log-rank test *P* value for active ABD as a predictor for ER. The *blue line* indicates the accumulated probability of ER recovery in patients with AMS 6 to 7 ABD before 4 months of age; the *red line* indicates the accumulated probability of ER recovery in patients with AMS 0 to 5 ABD at 4 months of age. Patients who are censored either did not achieve recovery of ER to AMS 6 by the end of the follow-up or underwent any of the following procedures: an anterior release surgery with subscapular tendon lengthening if needed because of restricted passive ER, SAN-SSN transfer with or without simultaneous anterior release and subscapular tendon lengthening if needed, and a tendon transfer operation with or without simultaneous anterior release and subscapular tendon lengthening if needed.

Patients were divided into 2 groups based on their shoulder ABD at 4 months: AMS 6 to 7 (group ABD 1) and AMS 0 to 5 (group ABD 2). Thirty-three patients had missing information regarding ABD before 4 months (Table 1). Sixty-seven percent (10 of 15) of patients in group ABD 1 and 53% (29 of 55) in group ABD 2 spontaneously recovered ER AMS 6. There was no difference between the groups ABD 1 and ABD 2 in the survival analysis (Fig. 4) (**see Table, Supplemental Digital Content 1**, http://links.lww.com/PRS/H688).

### Relationship between Narakas Classification at 1 Month and Recovery of ER

**Fig. 5.**
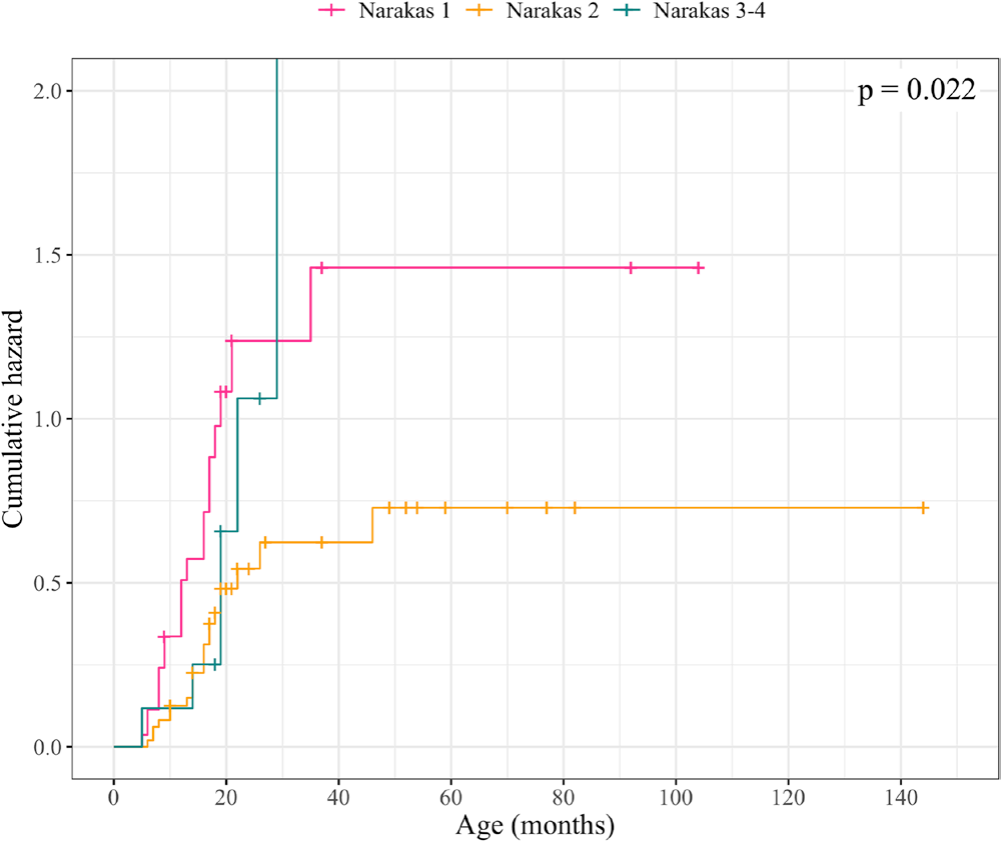
Kaplan-Meier hazard curves and log-rank test *P* value for Narakas grade at 1 month (Al-Qattan MM. Obstetric brachial plexus injuries. *J Am Soc Surg Hand* 2003;3:41–54) as a predictor for ER. Patients who are censored either did not achieve recovery of ER to AMS 6 by the end of the follow-up or underwent any of the following procedures: an anterior release operation, with subscapular tendon lengthening if needed because of restricted passive ER; SAN-SSN transfer, with or without simultaneous anterior release and subscapular tendon lengthening if needed; or a tendon transfer operation, with or without simultaneous anterior release and subscapular tendon lengthening if needed.

Seventy-one percent (20 of 28) of patients in group Narakas 1, 41% (21 of 51) in group Narakas 2, and 67% (6 of 9) in groups Narakas 3 and 4 spontaneously recovered active ER. Information regarding Narakas grade at 1 month was missing for 15 patients (Table 1). Groups Narakas 1 and Narakas 2 were significantly different in Kaplan-Meier analysis and univariate and multivariate Cox regression models (Fig. 5) (**see Table, Supplemental Digital Content 1**, http://links.lww.com/PRS/H688).

## DISCUSSION

In our consecutive cohort, 51% of the BPBI patients with absent ER still present at 3 months of age spontaneously recovered good active ER. There was a decline in the rate of spontaneous ER recovery at 2 years of age, after which spontaneous recovery of ER was not likely. Two years of age can thus be considered as the meaningful upper limit for follow-up of spontaneous recovery of ER. Early active EF was associated with spontaneous recovery of active ER, but early active ABD of the shoulder was not. Our findings suggest that in patients with no indication for early surgical treatment before age of 6 months, spontaneous recovery of ER can be expected up to 2 years, but when considering the optimal timing for a decision about possible peripheral nerve transfer surgery, the relationship between muscle denervation time and surgical outcome must also be taken into account.

The strength of this study is a large unselected consecutive patient cohort with good adherence to long-term follow-up. Some studies about conservative treatment outcomes exist,^1,4,5,7,8,17–21^ but to our knowledge, there are no studies about schedule of spontaneous recovery of ER in BPBI patients. However, a retrospective study design, heterogenic patient cohort, small number of certain injury types, and some missing information made it difficult to compare the spontaneous recovery outcomes based on the type of injury. Furthermore, the type and grade of BPBI—description of the affected roots and classification of injury to axonotmesis, neurotmesis, or root avulsion—was not included in the analysis, because determination of these can be very difficult and inaccurate, particularly without exploration in conservatively treated patients.^5,22^ In addition, examination and grading of individual muscle activity in young children can be difficult. Evaluations were based on a visual estimation, which is the only feasible practice. To reduce inaccuracy in the measurements, we used both a physician’s and a physiotherapist’s independent assessments and records. Also, as the true time point for recovery to AMS 6 usually took place between clinical evaluations, there was some inaccuracy in the time points for recovery to AMS 6, and this translates into a later time point of recovery of active movement. However, based on our sensitivity analysis, which had the maximum opposite (early recovery) bias with time points, we confirmed that the method for estimating the time point for ER recovery to AMS 6 does not substantially influence the results.

When our results are compared with previous reports of complete spontaneous recovery of ER after BPBI,^1,5,6^ the proportion of patients who recovered active ER was slightly worse in our cohort of 103 patients. This is probably because patients with a minor injury and full recovery of ER within the first 3 months of life were excluded from our study. If the patients with early spontaneous recovery are also considered, spontaneous recovery of ER to AMS 6 was observed in 73%, which is in line with the previous reports.

There is no consensus on the optimal timing for making the decision about—or performing—surgical procedures for those BPBI patients who do not spontaneously recover ER.^9,10,23^ Our results show that spontaneous recovery of ER can be expected until the age of 2 years. It must be noted that the patients in our study with recovery of ER after 2 years were evaluated during the beginning of our study period when SAN-SSN transfer surgery had not yet become an established practice, and thus, the systematic evaluation of shoulder muscle functions was performed less systematically during the early years. In addition, the use of BTX injections has enabled maintaining passive ER and shoulder congruency while waiting for the recovery of active movement. Early recognition and treatment of possible restriction of passive range of shoulder movement seems important to provide for unobstructed recovery of active ER.^15^ Morphologic defects progress over time if the muscle forces are absent,^22,24,25^ and glenohumeral joint incongruence can be detected as early as 2 months of age.^22,25^ In turn, deterioration of ER function in some patients could have been attributable to insufficient recovery of the nerve, which may lead to inadequate development of affected muscles and joint structures when the upper extremity grows in size. The majority of these patients had initially recovered ER AMS 6 after 1 year of age.

In our study, early EF function was significantly associated with recovery of active ER, which is in agreement with previous reports by Waters and Smith et al.^8,18^ In contrast, Xu et al. reported that none of the patients with no EF at 3 months of age recovered functional active ER, but their retrospective study included only patients who had already been treated in other hospitals for 3 to 4 years with no recovery.^19^ In contrast, early active shoulder ABD did not correlate with recovery of active ER, which has also been suggested by Michelow et al.^7^ Patients with Narakas grade 1 at 1 month recovered ER more often when compared with patients with Narakas grade 2. Because of a small number of patients with Narakas grade 3 or 4, no further conclusions can be drawn about their recovery compared with the other groups. Nevertheless, functional recovery of BPBI patients may be incorrectly predicted if only 1 prognostic parameter is used.^7^

Lack of standard outcome variables has led to a variety of different outcomes in studies on BPBI patients, and that prevents pooling and comparing specific outcomes from different studies. Furthermore, pooled results can be misleading, particularly because of differences in patient referral indications, age at the first clinical evaluation, and definitions or determination of reported variables.^14^ Further research is needed on recovery of shoulder function in BPBI patients to determine the optimal combination of prognostic factors for spontaneous recovery of ER and on the relationship between timing of possible peripheral nerve transfer (SAN-SSN transfer) and its outcome. This would help to determine the optimal age for making a decision about possible peripheral nerve transfer within the 2-year window of expected spontaneous recovery of ER.

## DISCLOSURE

The authors have no potential conflicts of interest with respect to the research, authorship, or publication of this article.

## Supplementary Material


