# Supplementary material for: Spontaneous Recovery of Active Shoulder External Rotation in Patients with Brachial Plexus Birth Injury
Source: Plast Reconstr Surg. 2024 Nov 20;155(6):1045–53. doi: 10.1097/PRS.0000000000011877 (PMC12105953; doi:10.1097/PRS.0000000000011877)
Supplement: Supplementary file 1 [file prs-155-1045e-s001.pdf]

Table, Supplemental Digital Content 1. Cox proportional hazards regression for spontaneous recovery of shoulder external rotation (ER).

		CHR	95% CI	*p*-value	AHR	95% CI	*p*-value
**Elbow flexion at 4 months**				0.036*			0.035*
	**AMS 0–5**	1	–	–	1	–	–
	**AMS 6–7**	1.81	1.04–3.14	0.036*	1.69	0.79–3.61	0.178
**Shoulder abduction at 4 months**				0.349			0.558
	**AMS 0–5**	1	–	–	1	–	–
	**AMS 6–7**	1.42	0.69–2.94	0.335	0.61	0.25–1.46	0.263
**Narakas at 1 month**				0.029*			0.100
	**Narakas 1**	1	–	–	1	–	–
	**Narakas 2**	0.43	0.23–0.80	0.007*	0.43	0.20–0.93	0.032*
	**Narakas 3 or 4**	0.71	0.28–1.78	0.463	0.48	0.13–1.78	0.271

AMS = Active Movement Scale; CHR = crude hazard ratio; CI = confidence interval; AHR = adjusted hazard ratio
